# Hypercalcemia as a rare presentation of angioimmunoblastic T cell lymphoma: a case report

**DOI:** 10.1186/s13256-018-1669-0

**Published:** 2018-04-20

**Authors:** Sana Chams, Inaya Hajj Hussein, Skye El Sayegh, Nour Chams, Khalid Zakaria

**Affiliations:** 1 Department of Internal Medicine, Wayne State University School of Medicine, Rochester Hills, MI USA; 2 Department of Biomedical Sciences, Oakland University William Beaumont School of Medicine, Rochester, MI USA

**Keywords:** Angioimmunoblastic T cell lymphoma, AITL, Hypercalcemia

## Abstract

**Background:**

Angioimmunoblastic T cell lymphoma is a rare malignancy, accounting for only 2% of all non-Hodgkin lymphomas, first described in the 1970s and subsequently accepted as a distinct entity in the current World Health Organization classification. Due to the paucity of this disease, there is still no identifiable etiology, no consistent risk factors, and the pathogenesis remains unclear.

**Case presentation:**

An 83-year-old Caucasian man presented to an emergency department with palpitations and was found to have atrial fibrillation. During his hospitalization, he was found to have asymptomatic hypercalcemia with corrected calcium of 11.7. Ten days later while in rehabilitation, he started complaining of progressive fatigue and altered mental status was noted. He was found to have a calcium level of 15.5 and was admitted to the intensive care unit for management and further workup. He was found at that time to have, parathyroid hormone: < 1; 25 hydroxyvitamin D: 74; 1,25 dihydroxyvitamin D: 85.4; angiotensin-converting enzyme: 7; parathyroid hormone-related protein: < 2; and multiple myeloma workup was negative. Computed tomography of his chest and abdomen showed extensive retroperitoneal, pelvic, and mesenteric lymphadenopathy in addition to findings suggestive of peritoneal carcinomatosis. A right axillary lymph node biopsy showed immunohistochemical parameters consistent with angioimmunoblastic T cell lymphoma. After a lengthy discussion with his family, it was decided that no further treatment would be pursued. He had an aggressive course at the hospital during which he developed pleural effusions, ascites, and diffuse petechiae within 2 weeks; these were complications from his malignancy. Considering the poor outcomes of his aggressive disease, he decided to enroll in an out-patient hospice. He died within a few months as a result of cardiorespiratory arrest.

**Conclusions:**

This case illustrates a rare presentation of an extremely rare disease; that is, hypercalcemia in a patient who was later found to have angioimmunoblastic T cell lymphoma. Diagnosing angioimmunoblastic T cell lymphoma might be the most challenging part due to the wide array of clinical presentations, of which hypercalcemia accounts for only 1%. As seen in this case, most patients present in advanced stages of the disease with poor prognosis.

## Background

Angioimmunoblastic T cell lymphoma (AITL) is a rare malignancy that was first described in the 1970s and was subsequently accepted as a distinct entity in the current World Health Organization classification. AITL accounts for only 2% of all non-Hodgkin lymphomas but is considered the most common subtype (15–20%) of peripheral T cell lymphomas. Due to the paucity of this disease, there is still no identifiable etiology, no consistent risk factors, and the pathogenesis remains unclear. AITL is considered a disease of the elderly since most patients were diagnosed in their sixth and seventh decades (median age 59–64 years). Also, almost all of these patients presented in advanced stages of the disease (stages III–IV). Presenting signs and symptoms include mainly B symptoms, generalized lymphadenopathy, and splenomegaly/hepatomegaly (Table 1) [1–3]. Diagnosis can only be achieved through biopsy and histological examination of one of the enlarged lymph nodes. Specific laboratory findings seen in AITL mainly include elevated lactate dehydrogenase (LDH), anemia, hypergammaglobulinemia, and bone marrow involvement (Table 2) [4]. Since it is a very rare malignancy, physicians should have a high index of suspicion and an experienced pathologist involved to identify the characteristic morphological features to be able to diagnose AITL.

**Table 1:** Presenting symptoms and signs in angioimmunoblastic T cell lymphoma

Symptoms and signs	Frequency (% of patients)
B symptoms	68–85
Generalized lymphadenopathy	94–97
Splenomegaly	70–73
Hepatomegaly	52–72
Skin rash	48–58
Polyarthritis	18
Ascites/effusions	23–37

Data grouped from Tobinai *et al.* [1], Siegert *et al.* [2], and Pautier *et al.* [3]

**Table 2:** Laboratory findings in angioimmunoblastic T cell lymphoma

Laboratory findings	Frequency (%, *n* = 243)
Elevated LDH	60
Elevated C-reactive protein	35
Anemia	33
Hypergammaglobulinemia	30
Thrombocytopenia	25
Elevated beta 2-microglobulin	22
Hemolytic anemia	13
Hypercalcemia	1

*LDH* lactate dehydrogenase. Data grouped from Federico *et al.* [4]

In our case report, we discuss an 83-year-old Caucasian man who presented to an emergency department with palpitations and was found to have atrial fibrillation and asymptomatic hypercalcemia. He was finally diagnosed as having AITL. Our case was unusual in demonstrating hypercalcemia as a rare presentation of an extremely rare disease, AITL. The mechanism of hypercalcemia in this patient with AITL was not related to parathyroid hormone-related protein (PTHrP) which is the more common pathway in hypercalcemia of malignancy. Also, this case illustrates a very aggressive course of AITL leading to the death of our patient within only a few months after he opted for hospice care. We followed CARE reporting guidelines in publishing our case report with important information from our case presented as a timeline (Table 3).

**Table 3:** Timeline table

Relevant past medical history and interventions		
Past medical history significant for coronary artery disease status post one stent with new onset atrial fibrillation.		
Summaries from initial and follow-up visits	Diagnostic testing	Interventions
During hospitalization, patient started complaining of progressive fatigue and altered mental status was noted. The patient was found to have a calcium level of 15.5 mg/dL (8.6–10.2 mg/dL). CT of the abdomen with contrast was suggestive of peritoneal carcinomatosis. Morphological and immunohistochemical findings from axillary lymph node biopsy were found to be consistent with angioimmunoblastic T cell lymphoma.	Laboratory studies: Ca; PTH; 25 hydroxyvitamin D; 1,25 dihydroxyvitamin D; ACE; PTHrP; and multiple myeloma workup.	After long discussions with the patient’s family, the decision was made for no further treatment.
After long discussions with the patient’s family, the decision was made for no further treatment. The patient had a complex hospital course in which he developed pleural effusions, ascites, and diffuse petechiae within 2 weeks; these were complications from his malignancy.	Imaging: abdominal CT. Lymph node biopsy: morphology, immunohistochemical study, and flow cytometry.	

*ACE* angiotensin-converting enzyme, *Ca* calcium, *CT* computed tomography, *PTH* parathyroid hormone, *PTHrP* parathyroid hormone-related protein

## Case presentation

**Fig. 1:**
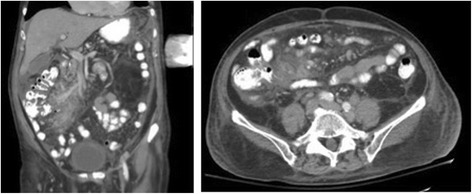
Computed tomography of the abdomen and pelvis. Coronal and axial computed tomography images with contrast of the patient’s abdomen showing extensive retroperitoneal, pelvic, and mesenteric lymphadenopathy in addition to extensive peritoneal and omental thickening with ascites

An 83-year-old Caucasian man with a past medical history significant for hypertension and coronary artery disease status post one stent presented to an emergency department with palpitations and was found to have atrial fibrillation. He is a retired building owner who lives with his spouse in a suburban area and enjoys gardening and model building as part of his hobbies. He does not smoke tobacco, he consumes alcohol occasionally, and he does not use illicit drugs. His family history is significant for colon cancer in his mother and hypertension and heart failure in his father. Prior to admission, he was on aspirin 81 mg daily, atorvastatin 40 mg daily, lisinopril 5 mg daily, and metoprolol 12.5 mg twice daily. On presentation: temperature 36.39 °C (97.5 °F), pulse 110 beats per minute, blood pressure 118/62 mmHg, respiratory rate 20 breaths per minute, and oxygen saturation of 98% on room air. On physical examination, he was awake, alert, and oriented to self, others, time, and place. His skin was warm, dry, with no apparent rashes. His neck was supple and non-tender with no jugular venous distension or apparent masses. A cardiovascular examination was significant for an irregularly irregular rhythm and rapid pulse, but no murmurs or gallops. He did not demonstrate any lower extremity edema and pulses were intact bilaterally. His lungs were clear with equal breath sounds. His abdomen was soft and non-tender with no hepatosplenomegaly. No lymphadenopathy was appreciated. A neurological examination showed grossly intact cranial nerves 2–12, normal sensation, strength was full bilaterally, normal reflexes, intact coordination, and normal gait. During his hospitalization, he underwent transesophageal echocardiography with cardioversion, which converted his irregular rhythm back to sinus rhythm. He was subsequently started on sotalol 80 mg twice daily and apixaban 5 mg twice daily. He was found to have asymptomatic hypercalcemia with corrected calcium of 11.7 mg/dL (8.6–10.2 mg/dL) incidentally. As part of the workup for his hypercalcemia, blood samples were sent for evaluation including vitamin D and parathyroid hormone (PTH) levels. He was sent to in-patient rehabilitation. Ten days into his stay in rehabilitation, he started complaining of progressive fatigue and altered mental status was noted. He was found to have a calcium level of 15.5 mg/dL (8.6–10.2 mg/dL) and was admitted to the intensive care unit for management and further workup. He was managed with aggressive fluid hydration, pamidronate 90 mg administered intravenously once, and calcitonin 300 U administered subcutaneously twice daily for a total of 2 days. He was found at that time to have: PTH, < 1 pg/mL (15–65 pg/mL); 25 hydroxyvitamin D, 74 ng/mL (30–100 ng/mL); 1,25 dihydroxyvitamin D, 85.4 pg/mL (19.9–79.3 pg/mL); angiotensin-converting enzyme (ACE), 7 U/L (9–67 U/L); and multiple myeloma workup was negative. PTHrP was also tested in order to identify the cause of hypercalcemia and was found to be undetectable: < 2.0 pmol/L (0.0–2.3 pmol/L). Our patient had a low LDH 131 U/L (reference range 135–225 U/L), bilirubin total was 0.55 mg/dL (reference range 0–1.2 mg/dL), and indirect bilirubin was 0.35 mg/dL (reference range 0.2–1.2 mg/dL). In addition, a peripheral smear showed mild normocytic normochromic anemia, with no microcytosis, favoring anemia of chronic disease/inflammation, thus our patient did not have evidence of hemolytic anemia. Hypergammaglobulinemia was evident with gamma globulin level of 2.64 mg/dL (reference range 0.62–1.51 mg/dL). Computed tomography (CT) of his abdomen with contrast showed extensive retroperitoneal, pelvic, and mesenteric lymphadenopathy in addition to extensive peritoneal and omental thickening with ascites suggestive of peritoneal carcinomatosis (Fig. 1).

**Fig. 2:**
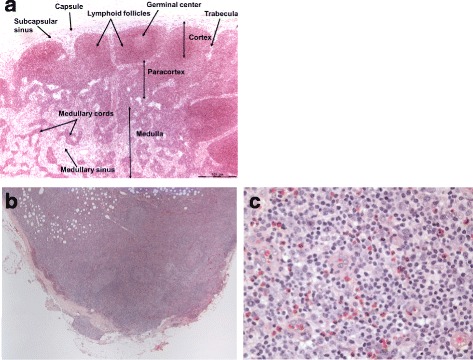
Hematoxylin and eosin staining of the lymph node. **a** Normal lymph node histology. **b** Lymph node biopsy obtained from patient showing architectural disarray with expansion of paracortical region, extracapsular extension, and lymphoid tissue bypassing the capsules as seen in low-power view. **c** Lymph node biopsy obtained from patient showing increase in eosinophils, increase in number of blood vessels (high endothelial venules), and a polymorphous infiltrate of small lymphocytes, plasma cells, and occasional immunoblasts as seen in high-power view

He underwent a right axillary lymph node biopsy, which initially revealed findings suspicious of T cell lymphoma. At the pathologist’s request, the slides were sent for consultation to a tertiary referral center. Hematoxylin and eosin (H&E) stained sections (Fig. 2) showed mildly enlarged lymph nodes with paracortical expansion by a polymorphous infiltrate of small lymphocytes, eosinophils, plasma cells, and occasional immunoblasts. Such findings are suggestive of a T cell lymphoma.

A subset of the small lymphocytes had a slightly increased amount of pale cytoplasm with reactive germinal centers. Occasional secondary follicles were also present. Immunohistochemical staining showed that the paracortical lymphocytes with slightly increased pale cytoplasm were positive for cluster of differentiation (CD) 2, CD3, CD4, CD5, CD7, and programmed cell death protein 1 (PD-1), with weak expression of CD10 and B cell lymphoma 6 protein (BCL6) in a subset. CD8 highlighted a minor subset of the small T cells; CD20 highlighted B cell follicles and increased paracortical immunoblasts. CD21 highlighted follicular dendritic cells (FDCs) associated with the B cell follicles and some of the high endothelial venules in the paracortex. Flow cytometry detected a small atypical T cell population with some co-expression of CD10 and a CD4/CD8 ratio of 4.5:1. T cell receptor (TCR) gene rearrangement detected both TCR-beta and TCR-gamma clonal rearrangements. Thus, the morphological and immunohistochemical findings were found to be consistent with AITL.

After long discussions with our patient’s family, the decision was made for no further treatment. He had a complex hospital course in which he developed pleural effusions, ascites, and diffuse petechiae within 2 weeks; these were complications from his malignancy. Considering the poor outcomes of his aggressive disease, our patient decided to enroll in an out-patient hospice with no further follow-up or intervention. He died within a few months as a result of cardiorespiratory arrest.

## Discussion

This is a case of an 83-year-old man who presented with atrial fibrillation, was incidentally found to have asymptomatic hypercalcemia, and was subsequently diagnosed as having AITL. This is an unusual case that demonstrates hypercalcemia as a rare presentation of an extremely rare disease, AITL. It also illustrates a very aggressive course of AITL leading to the death of our patient within only a few months after he opted for hospice care.

AITL is a rare and aggressive malignancy that is almost always diagnosed in patients when it has already reached advanced stages. The most common presenting signs and symptoms of AITL include generalized lymphadenopathy (94–97%), B symptoms (weight loss, fever, night sweats and so on; 68–85%), and splenomegaly (70–73%). On presentation, our patient did not have any B symptoms and no lymph nodes or splenomegaly were appreciated on physical examination. The most common laboratory findings in AITL include elevated LDH (60%), elevated C-reactive protein (CRP; 35%), anemia (33%), and hypergammaglobulinemia (30%). Our case illustrates hypercalcemia as a rare presentation of an extremely rare disease. Diagnosing AITL might be the most challenging part due to the wide array of nonspecific clinical presentations, of which hypercalcemia accounts for only 1% [4]. The mechanism behind hypercalcemia in a patient with AITL is yet to be identified. PTHrP is the principal factor in cancer-induced bone disease and is responsible for 80% of humoral hypercalcemia of malignancy and localized osteolysis associated with metastatic cancer; however, it was effectively excluded as the culprit in this case [5]. Our patient had an elevated 1,25 dihydroxyvitamin D with normal 25 hydroxyvitamin D and low PTH, which means that the hypercalcemia was caused by the increased production of 1,25 dihydroxyvitamin D through the humoral hypercalcemia of malignancy pathway [6, 7]. The hypercalcemia did not suppress, in this case, the production of 1,25 dihydroxyvitamin D through the suppression of PTH, as would happen in a normal physiologic response. This lack of suppression indicates that the production of 1,25 dihydroxyvitamin D is due to an extra-renal PTH-independent production. This ectopic production of 1,25 dihydroxyvitamin D remains unclear in lymphomas; however, proposed theories from various case studies relate it to production from tumor-associated macrophages or the malignant lymphocytes themselves [6, 7].

The index of suspicion among physicians for AITL is usually low given the wide array of clinical presentations and the lack of specific laboratory findings, with the diagnosis being made through biopsy only. A lymph node biopsy of AITL is characterized by the polymorphic infiltrate that leads to partial effacement of the lymph node’s architecture and predominantly occupies the paracortical area. The architectural changes in AITL follow three overlapping patterns. Pattern 1, seen in 20% of the patients, has preserved lymph node architecture with the cortex showing hyperplastic B cell follicles with poorly developed mantle zones. Pattern 2, as seen in this patient and in 30% of the patients, is characterized by a loss of normal architecture of the lymph node and the presence of concentrically arranged FDCs. Pattern 3, seen in 50% of the patients, has complete effacement of lymph node architecture, absent B cell follicles, and prominent irregular proliferation of FDCs. All three patterns have polymorphic infiltrates. The proliferation of FDCs in AITL is best appreciated through immunostaining of the FDC markers (CD21, CD23, CD35) with most, if not all, tumor cells in AITL expressing CD10. AITL follows an aggressive clinical course despite conventional chemotherapy with median survival being less than 3 years with a 10–30% survival rate at 5 years from diagnosis. Two thirds of patients can achieve complete remission; however, they often succumb to infectious complications. Therapies used to treat individuals with AITL include corticosteroids and both single agent regimens and combined chemotherapeutic regimens such as: cyclophosphamide, hydroxydaunomycin, Oncovin (vincristine), and prednisone (CHOP); cyclophosphamide, vincristine, and prednisone (CVP); and vincristine, asparaginase, and prednisone (VAP). One promising therapy for the treatment of AITL is high-dose chemotherapy followed by autologous stem cell transplantation [8, 9].

## Conclusions

This case illustrates a rare presentation of an extremely rare disease; that is, hypercalcemia in a patient who was later found to have AITL. Diagnosing AITL might be the most challenging part due to the wide array of clinical presentations of which hypercalcemia accounts for only 1%. As seen in this case, most patients present in advanced stages of the disease with poor prognosis. AITL often runs an aggressive course with dismal outcomes and a 5-year survival rate of only 30–35%. Despite the rarity of this malignancy, clinicians should be aware of its existence.

## Abbreviations

ACE: Angiotensin-converting enzyme
AITL: Angioimmunoblastic T cell lymphoma
BCL6: B cell lymphoma 6 protein
CD: Cluster of differentiation
CHOP: Cyclophosphamide, hydroxydaunomycin, Oncovin (vincristine), and prednisone
CRP: C-reactive protein
CT: Computed tomography
CVP: Cyclophosphamide, vincristine, and prednisone
FDCs: Follicular dendritic cells
H&E: Hematoxylin and eosin
LDH: Lactate dehydrogenase
PD-1: Programmed cell death protein 1
PTH: Parathyroid hormone
PTHrP: Parathyroid hormone-related protein
TCR: T cell receptor
VAP: Vincristine, asparaginase, and prednisone

## References

[1] Tobinai K, Minato K, Ohtsu T, Mukai K, Kagami Y, Miwa M, Watanabe S, Shimoyama M. Clinicopathologic, immunophenotypic, and immunogenotypic analyses of immunoblastic lymphadenopathy-like T-cell lymphoma. Blood. 1988;72:1000–6. 3046680

[2] Siegert W, Agthe A, Griesser H, Schwerdtfeger R, Brittinger G, Engelhard M, Kuse R, Tiemann M, Lennert K, Huhn D. Treatment of angioimmunoblastic lymphadenopathy (AILD)-type T-cell lymphoma using prednisone with or without the COPBLAM/IMVP-16 regimen. A multicenter study. Kiel Lymphoma Study Group. Ann Intern Med. 1992;117:364–70. 10.7326/0003-4819-117-5-364 1380221

[3] Pautier P, Devidas A, Delmer A, Dombret H, Sutton L, Zini JM, Nedelec G, Molina T, Marolleau JP, Brice P. Angioimmunoblastic-like T-cell non-Hodgkin’s lymphoma: outcome after chemotherapy in 33 patients and review of the literature. Leuk Lymphoma. 1999;32:545–52. 10.3109/10428199909058412 10048427

[4] Federico M, Rudiger T, Bellei M, *et al*. Clinicopathologic Characteristics of Angioimmunoblastic T-Cell Lymphoma: Analysis of the International Peripheral T-Cell Lymphoma Project. J Clin Oncol. 2013;31(2):240–6. 10.1200/JCO.2011.37.3647 PMC3532394 22869878

[5] Mundy G, Edwards J. PTH-Related Peptide (PTHrP) in Hypercalcemia. J Am Soc Nephrol. 2008;19(4):672–5. 10.1681/ASN.2007090981 18256357

[6] Hewison M, Kantorovich V, Liker H, *et al*. Vitamin D-Mediated Hypercalcemia in Lymphoma: Evidence for Hormone Production by Tumor-Adjacent Macrophages. J Bone Miner Res. 2003;18(3):579–82. 10.1359/jbmr.2003.18.3.579. 12619944

[7] Tebben P, Singh R, Kumar R. Vitamin D-Mediated Hypercalcemia: Mechanisms, Diagnosis, and Treatment. Endocr Rev. 2016;37(5):521–47. 10.1210/er.2016-1070. PMC5045493 27588937

[8] Dogan A, Attygalle A, Kyriakou C. Angioimmunoblastic T-cell lymphoma. Br J Haematol. 2003;121(5):681–91. 10.1046/j.1365-2141.2003.04335.x 12780782

[9] Iannitto E, Ferreri A, Minardi V, Tripodo C, Kreipe H. Angioimmunoblastic T-cell lymphoma. Crit Rev Oncol Hematol. 2008;68(3):264–71. 10.1016/j.critrevonc.2008.06.012 18684638

